# Toxicity assessment of zinc oxide nanoparticles using sub-acute and sub-chronic murine inhalation models

**DOI:** 10.1186/1743-8977-11-15

**Published:** 2014-04-01

**Authors:** Andrea Adamcakova-Dodd, Larissa V Stebounova, Jong Sung Kim, Sabine U Vorrink, Andrew P Ault, Patrick T O’Shaughnessy, Vicki H Grassian, Peter S Thorne

**Affiliations:** 1 Department of Occupational and Environmental Health, University of Iowa, Iowa City, IA 52242, USA; 2 Department of Chemistry, University of Iowa, Iowa City, IA 52242, USA

**Keywords:** Zinc oxide nanoparticles, Dissolution, Inhalation, Murine model, Pulmonary response, Toxicity

## Abstract

**Background:**

Although ZnO nanoparticles (NPs) are used in many commercial products and the potential for human exposure is increasing, few *in vivo* studies have addressed their possible toxic effects after inhalation. We sought to determine whether ZnO NPs induce pulmonary toxicity in mice following sub-acute or sub-chronic inhalation exposure to realistic exposure doses.

**Methods:**

Mice (C57Bl/6) were exposed to well-characterized ZnO NPs (3.5 mg/m^3^, 4 hr/day) for 2 (sub-acute) or 13 (sub-chronic) weeks and necropsied immediately (0 wk) or 3 weeks (3 wks) post exposure. Toxicity was assessed by enumeration of total and differential cells, determination of total protein, lactate dehydrogenase activity and inflammatory cytokines in bronchoalveolar lavage (BAL) fluid as well as measurements of pulmonary mechanics. Generation of reactive oxygen species was assessed in the lungs. Lungs were evaluated for histopathologic changes and Zn content. Zn concentration in blood, liver, kidney, spleen, heart, brain and BAL fluid was measured.

**Results:**

An elevated concentration of Zn^2+^ was detected in BAL fluid immediately after exposures, but returned to baseline levels 3 wks post exposure. Dissolution studies showed that ZnO NPs readily dissolved in artificial lysosomal fluid (pH 4.5), but formed aggregates and precipitates in artificial interstitial fluid (pH 7.4). Sub-acute exposure to ZnO NPs caused an increase of macrophages in BAL fluid and a moderate increase in IL-12(p40) and MIP-1α, but no other inflammatory or toxic responses were observed. Following both sub-acute and sub-chronic exposures, pulmonary mechanics were no different than sham-exposed animals.

**Conclusions:**

Our ZnO NP inhalation studies showed minimal pulmonary inflammation, cytotoxicity or lung histopathologic changes. An elevated concentration of Zn in the lung and BAL fluid indicates dissolution of ZnO NPs in the respiratory system after inhalation. Exposure concentration, exposure mode and time post exposure played an important role in the toxicity of ZnO NPs. Exposure for 13 wks with a cumulative dose of 10.9 mg/kg yielded increased lung cellularity, but other markers of toxicity did not differ from sham-exposed animals, leading to the conclusion that ZnO NPs have low sub-chronic toxicity by the inhalation route.

## Introduction

Materials engineered at the nanoscale currently exist in over a thousand consumer products including cosmetics, electronics, medical devices, and textiles [1]. Metal and metal oxide nanoparticles (NPs) are also under development for antimicrobial, self-decontaminating and UV blocking functions for both military protection gear and civilian health products. Ag, TiO_2_, ZnO, and CeO_2_ are among the nanomaterials most widely incorporated into market goods. Zinc oxide (ZnO) NPs are used in various product categories because of their unique photocatalytic, electronic, optical, dermatological, and antibacterial properties; however the most common applications are in sunscreens [2], baby powders, anti-dandruff shampoos, and fabric treatments for UV shielding [3,4]. Welding fumes, especially from galvanized steel, can also be source of exposure to ZnO aerosol in the nanometer range [5,6]. Additionally, ZnO NPs have received more attention due to their putative anti-cancer properties and for drug delivery [7].

Most of the production processing takes place within closed systems, however during postproduction and packaging, ZnO NPs may be released into the ambient air where they circulate for some time. During this time there is a potential for worker and public exposure *via* inhalation [2]. With increasing interest to their potential toxicity, adverse effects of ZnO NPs have been recently studied *in vivo*[8-13] and *in vitro*[14-21]. However, despite the growing literature on nanomaterials applications, the information about biological effects of ZnO NPs is still insufficient and often controversial. Many reports using *in vitro* systems indicate that the mechanism of ZnO toxicity involves the generation of reactive oxygen species (ROS) [15,16,18,22]. Some report that dissolution of ZnO, which is enhanced for the smallest particles [23] plays an important role in the toxicity mechanism of ZnO NPs [18,19,22]. It has been shown that ZnO dissociation disrupts cellular zinc homeostasis in mouse leukemic monocyte macrophage cells (RAW 264.7), leading to lysosomal and mitochondria damage and ultimately cell death [22]. Another *in vitro* study indicated that free Zn^2+^ ions are not a major contributor of ROS generation [16]. The release of ions from ZnO NPs in biological media depends on many factors, such as pH, ligands present in the solution, surface groups, or impurities [11]. Because of these effects, it can be lower or higher than predicted from aqueous phase thermodynamic behavior of ZnO alone [18]. A limitation of the above-referenced studies is that the nanoparticle dose used usually exceeds an environmentally relevant dose. Moreover, these *in vitro* models cannot replicate the intact cardiovascular system and various cellular interactions present in the body. Hence, *in vitro* models fall short of accurately predicting the toxicological behavior of the nanoparticles in living organisms, especially if studied in submersed conditions when particles are suspended in media [24] which can impact dispersion and dissolution.

More recently, there is an increasing body of literature reporting on ZnO NP toxicity studies *in vivo*[8-12,25]. An oral exposure of mice to 20 and 120 nm particles indicated minimal toxicity of ZnO NPs, where smaller NPs were slightly more toxic than the larger ones [9]. Some pathological damage was observed in gastric, liver, heart and spleen in this oral toxicity study. Pulmonary toxicity has been assessed in instillation [8,11,12,26] and inhalation exposure [10] models. Instillation studies in rodents have shown that ZnO NPs are capable of inducing acute pulmonary inflammation, including neutrophil recruitment and lactate dehydrogenase (LDH) release at cumulative doses of 0.6 mg/kg [11] and 5 mg/kg [12]. Moreover, it has been suggested that metal oxide NPs may produce unique inflammatory footprints in the lung depending on the metal content and a propensity to deliver soluble ions [8]. ZnO NPs exhibited eosinophilic inflammation without cytotoxicity in the rat instillation exposure study by Cho *et al*. [8]. A decreased dissolution of ZnO NPs by iron doping reduced pulmonary inflammation in another oropharyngeal and intratracheal instillation rat study [11].

In order to better understand, and potentially resolve, disparate findings from *in vivo* instillation studies and *in vitro* studies, we exposed male C57Bl/6 mice to fully characterized commercially available ZnO NPs by inhalation in a whole-body inhalation chamber for periods of 2 or 13 wks. The potential toxic effects associated with the inhalation of ZnO NPs were assessed in mice with evaluation of lung inflammation, cytotoxicity, oxidative stress, pulmonary mechanics with methacholine challenge and hematology parameters. Body burden of zinc in the lungs, blood and other selected tissues was measured.

## Materials and methods

### Nanomaterial bulk properties characterization

Zinc oxide NPs with stated primary particle average diameter of 10 nm were purchased in two different lots (Meliorum Technologies, Inc. Rochester, NY) and used as received. Powder X-ray diffraction (XRD) was performed using Bruker D-5000 q – q X-ray diffractometer with Kevex-sensitive detector (Madison, WI) to identify crystalline phases present in the sample. We assessed the primary particle size of 400 random ZnO NPs by transmission electron microscopy (TEM) (JEOL JEM-1230, Japan) to evaluate the veracity of the manufacturer’s specifications, as well as to image the NPs aerosols generated in the inhalation exposure chamber.

Surface area and surface composition of the ZnO NPs were measured. For surface area measurements, an automated multipoint BET surface area apparatus (Quantachrome Nova 4200e, Boynton Beach, FL) using nitrogen gas as the adsorbent was used. Samples were degassed at 100°C for 12 hr under vacuum prior to the analysis. Surface composition was characterized using attenuated total reflectance (ATR) Fourier transform infrared (FTIR) spectroscopy equipped with a MCT/A detector (Thermo Scientific Nicolete FTIR spectrometer, Waltham, MA). For these measurements, a thin, evenly coated ZnO film was deposited onto an AMTIR (amorphous material transmitting infrared radiation) crystal element in a horizontal ATR cell (Pike Technologies, Inc.). The film was prepared by placing a suspension of ZnO NPs (1 mg/mL in Optima water) onto the crystal and drying overnight. X-ray photoelectron spectroscopy (XPS) was used to probe the surface chemical composition of the powdered sample (Ultra-Axis XPS, Kratos, Manchester, UK) as previously described [27].

### Dissolution measurements

Dissolution of ZnO NPs was measured in simulated biological fluids including artificial lysosomal fluid (ALF) (pH = 4.5) and Gamble’s solution (pH = 7.4) using inductively coupled plasma optical emission spectroscopy (ICP-OES) (Varian 720 ES, Walnut Creek, CA) as previously described [28]. The ALF fluid simulates the phagolysosomal composition and pH of alveolar and interstitial macrophages and the Gamble’s solution is used to mimic the interstitial fluid in the lungs.

### Animals

Inhalation exposure studies followed the same experimental design as in several of our previous studies [28-30]. Mice (C57Bl/6, males, 5 wks old, The Jackson Laboratory, Bar Harbor, ME) were acclimatized for 12 days before exposure while housed in our vivarium in polypropylene, fiber-covered cages in HEPA-filtered Thoren caging units (Hazelton, PA). Food (sterile Teklad 5% stock diet, Harlan, Madison, WI) and water (*via* an automated watering system) were provided *ad libitum.* Light–dark cycle (12 hr) was maintained in the animal room. All protocols were approved by the Institutional Animal Care and Use Committee at the University of Iowa.

Animals without restraint were exposed by inhalation for 4 hr/day, 5 days/wk for period of 2 wks in the sub-acute study and for 13 wks in the sub-chronic study. In both studies, mice were necropsied either within one hr (0 wk) or 3 wks after (3 wks) the last exposure. During the 3 wk period post exposure, animals remained in the vivarium without exposure. Both studies also included sentinel mice that were housed in the vivarium during the whole duration of each study. The average mass concentration of ZnO NPs in the whole-body chamber in sub-acute study was 3.6 ± 0.5 mg/m^3^ (mean ± standard deviation) and in the sub-chronic study was 3.3 ± 0.6 mg/m^3^. Control animals (shams) were exposed to HEPA-filtered laboratory air in the identical exposure chamber in an adjacent laboratory. We estimated cumulative doses of 51 and 306 μg/mouse (in sub-acute and sub-chronic study, respectively) assuming a minute volume of 25 mL/min and a deposition fraction of 0.24 in the tracheobronchial and pulmonary region. Deposition fraction was based on a rat computational fluid dynamic model [31] and an average particle size (40 nm) as measured by a scanning mobility particle sizer (SMPS).

### Generation of aerosol and inhalation exposure of mice

Mice in this study were exposed in a whole-body exposure chamber, as previously described [32]. ZnO aerosols were generated from a suspension of ZnO NPs in water (Optima grade, Fisher Scientific, Pittsburgh, PA) from a liquid concentration of 1 mg ZnO NPs/mL using a 6-jet Collison nebulizer (BGI Inc. Waltham, MA) supplied with filtered pressurized air. To minimize agglomeration, a suspension of ZnO NPs in water was prepared freshly each day and ultra-sonicated (continuous sonication) with a high frequency probe set at 30% of the maximum amplitude at 20 kHz (model 550, Fisher Scientific, Pittsburgh, PA) for 20 minutes before it was placed into the nebulizer. Most of our practices for ultrasonic dispersion of NP suspension were in an agreement with recommendations by Taurozzi *et al*. [33]. A magnetic stir bar was placed into the nebulizer to facilitate stirring of the ZnO suspension during nebulization. Aerosol generated by the nebulizer passed through a brass drying column heated to 110°C (to evaporate water droplets) as well as a tube containing a 20 mCi ^63^Ni source (to remove static charge) prior to entering the chamber.

To assess the size distribution of generated ZnO NP aerosol during animal exposure, the air from the chamber was sampled at flow 1 L/min using an SMPS (TSI Inc., Shoreview, MN, model 3036 with model 3081 long-differential analyzer and model 3785 water-based condensation particle counter, measuring particle diameters in 105 size channels between 7.4 - 289 nm). Geometric mean (GM) mobility diameter and geometric standard deviation (GSD) of aerosol sizes in individual exposures were calculated.

Mass concentration of the aerosol in the whole-body chamber was measured gravimetrically using 47-mm fiberglass filters (Whatman, Middlesex, UK) held in stainless steel holders placed in line with the exhaust air flow (24 L/min). Filters were pre- and post-weighed using a calibrated Mettler MT5 six-place balance (Mettler-Toledo, Inc., Columbus, OH) placed in a dedicated climate-controlled room and were changed every two hours. Generated aerosol in the chamber was collected on nickel grids placed inside the exposure chamber or using an electrostatic precipitator (Model 100, ESP Nano Inc., Spokane WA).

### Bronchoalveolar lavage fluid and blood analyses

Euthanasia of mice was performed by overdose of isoflurane followed by cervical dislocation and exsanguination through the heart. The lungs (n = 6 in each experimental group) were lavaged, *in situ*, 3 times with approximately 1 mL of 0.9% sterile sodium chloride solution (Baxter, Deerfield, IL), the recovered bronchoalveolar lavage (BAL) fluid was centrifuged (800 × g, 5 min), the cell pellet was used for enumeration of total and differential cell counts. The lavage supernatants were stored at -80°C for later analyses of total protein using Bradford protein assay (Bio-Rad Laboratories, Inc., Hercules, CA), LDH activity (Roche Diagnostics, Penzberg, Germany) and cytokine levels measured by multiplexed fluorescent bead-based immunoassays (Bio-Plex Pro Mouse Cytokine, Chemokine, and Growth Factor Multiplex Assays, Bio-Rad Laboratories, Inc., Hercules, CA). Measured cytokines in both studies included: interleukin (IL)-6, IL-12(p40), tumor necrosis factor (TNF)-α, granulocyte macrophage colony stimulating factor (GM-CSF), keratinocyte-derived cytokine (KC), monocyte chemotactic protein (MCP)-1, and macrophage inflammatory protein (MIP)-1α. In sub-chronic study, we also measured IL-4, IL-5, IL-13 and IL-17. Blood samples for analyses of hematology parameters (n = 6 in each group) were collected by cardiac puncture into tubes containing EDTA (Multivette®, SARSTEDT AG & Co., Nümbrecht, Germany) and analyzed using an automated hematology analyzer (Sysmex XT-2100, Kobe, Japan).

### Lipid peroxidation assay

To investigate the ZnO NP-mediated generation of ROS after sub-chronic exposure to ZnO NPs, malondialdehyde (MDA, product of lipid peroxidation) levels were measured in lung tissue homogenates (n = 6) using the thiobarbituric acid reactive substances (TBARS) assay kit (Cayman Chemical Company, Ann Arbor, MI) following the manufacturer’s protocol.

### ICP-MS elemental analysis of BAL fluid and organs

Cell-free BAL supernatants were centrifuged at 44,900 × g for 30 min and Zn^2+^ concentrations were determined. Lung, liver, spleen, kidney, heart and brain tissues from mice in each group were stored at −80°C immediately after resection. Tissues were lyophilized for 16 hr at 0.13 mBar and −50°C in a freeze dryer (Labconco Corp., Kansas City, MO) and then weighed. The tissues were digested in a HotBlock™ digestion system (Environmental Express, Mt. Pleasant, SC) at 95-98°C using mixtures of high purity concentrated hydrochloric acid and nitric acid (Fisher Optima® grade) in 1:3 ratio, respectively. Metal analysis of the digested tissues, blood and BAL fluid was performed using inductively coupled plasma mass spectrometer (Xseries 2 quadrupole ICP-MS, Thermo Fisher Scientific Inc., West Palm Beach, FL) with a method detection limit for Zn of less than 1 μg/kg. Each sample was spiked with cobalt as an internal standard at 20 μg/L.

### Pulmonary mechanics measurements

Airway hyper-reactivity to methacholine challenge in mice after exposure to ZnO nanoparticle aerosol (n = 6 in sub-acute study, n = 8 at 0 wk and n = 3 at 3 wks post exposure in sub-chronic study) was assessed using a forced oscillation technique in the FlexiVent system (SCIREQ, Montreal, QC, Canada). Anesthesia was induced by intraperitoneal administration of 90 mg/kg of pentobarbital sodium (Ovation Pharmaceuticals, Inc. Deerfield, IL, USA). The trachea was exposed and tracheotomy performed using a tracheal cannula with luer adapter (1.3 mm, length 20 mm, Harvard Apparatus, Holliston, MA, USA). The cannula was connected to a computer-controlled small animal ventilator set at a frequency of 150 breaths/min, a tidal volume of 10 mL/kg, and a positive end-expiratory pressure of 2 to 3 cm H_2_O. After measurement of a baseline, each mouse was challenged with increasing concentrations of methacholine aerosol (1, 3, 10, and 30 mg/mL) that were generated for 10 s with an in-line nebulizer directly through the canulated trachea. Dynamic resistance (R) and dynamic compliance (C) were measured using a “snapshot” protocol each 20 s for 5 min, ensuring that measured parameters stabilized. The mean of these 15 values was calculated for each methacholine dose. After each dose, the lungs were inflated to total lung capacity to return the measurements to baseline.

### Histopathology of lung tissues

Mice that were not lavaged (n = 5 and n = 6 in sub-acute and sub-chronic study, respectively) were perfused through the heart with saline and lungs were fixed in 10% buffered formalin *via* the cannulated trachea. The tissues were paraffin-embedded, sectioned at 5 μm, and stained with hematoxylin and eosin (H&E). Tissue sections were evaluated by a board-certified veterinary pathologist using light microscopy with the focus on parenchymal architecture (bronchioles, alveoli, pleura, and vasculature), and presence or absence of inflammatory cell infiltrates, evidence of acute lung injury, lymphoid agglomerates or fibrosis. We also evaluated BAL cells and lung tissues using dark field microscopy as well as TEM equipped with an energy dispersive spectrometer (EDS), as described previously [28].

### Statistical analyses

Outcome measures from mice exposed to ZnO NPs and control mice exposed to filtered laboratory air were analyzed using one-way analyses of variance (ANOVA) followed by Tukey test (Sigma Plot v.11.0, Systat Software Inc., Point Richmond, CA). If data were not normally distributed, Kruskal-Wallis ANOVA on Ranks was used. A *p*-value less than 0.05 was considered statistically significant. Data are expressed as mean ± standard error (SE) unless otherwise noted.

## Results

### ZnO nanoparticle characterization

**Figure 1:**
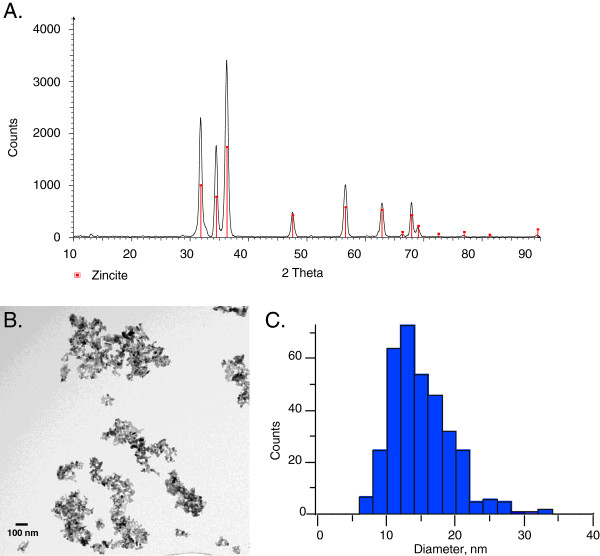
**Characterization of the phase and size of ZnO nanoparticles. A.** XRD spectrum pattern of the nanoparticles (black) compared to XRD spectrum of reference material, zincite (red lines). **B.** TEM image of ZnO nanoparticles deposited onto a TEM grid from a methanol solution. **C.** Particle size distribution determined from TEM.

The XRD pattern for the ZnO NPs are compared to standard crystalline zincite in Figure 1A. These data show that the nanoparticles have the same crystal structure as that of bulk zincite. Figure 1B shows a TEM image of nanoparticle agglomerates deposited on a TEM grid from a methanol solution. The diameters of individual particles were assessed by measurement of over 400 particles and yielded a primary particle size distribution of 15 ± 4 nm and 26 ± 11 nm for the two lots of NPs used in sub-acute and sub-chronic study, respectively (Figure 1C and Table 1).

**Table 1:** Summary of physicochemical properties of ZnO NPs and generated aerosol

	**ZnO NPs**	
Primary particle size^1^	15 ± 4 nm (26 ± 11 nm)^#^	
Crystalline or amorphous	Crystalline, zincite	
Surface functionality	CO_3_^2−^, OH^−^, CH	
BET surface area^2^	47 ± 1 m^2^/g (15 ± 4 m^2^/g)^#^	
Nanoparticle dissolution	Gamble’s solution (pH 7.4): < 1%	
	ALF* (pH 4.5): 100%	
Aerosol characterization in the whole-body exposure chamber:		
	**Sub-acute study**	**Sub-chronic study**
Aerosol size distribution^3^	GM = 46 nm	GM = 36 nm
	GSD = 1.8	GSD = 1.8
Exposure mass concentration^2^	3.6 ± 0.5 mg/m^3^	3.3 ± 0.6 mg/m^3^

^1^Primary diameter as measured by TEM in our laboratory ± standard deviation (SD), ^2^Mean ± standard deviation, ^3^GM: Geometric mean mobility diameter and GSD: geometric standard deviation, ^#^Analysis of ZnO NPs used in sub-chronic study in parenthesis. *ALF: artificial lysosomal fluid.

**Figure 2:**
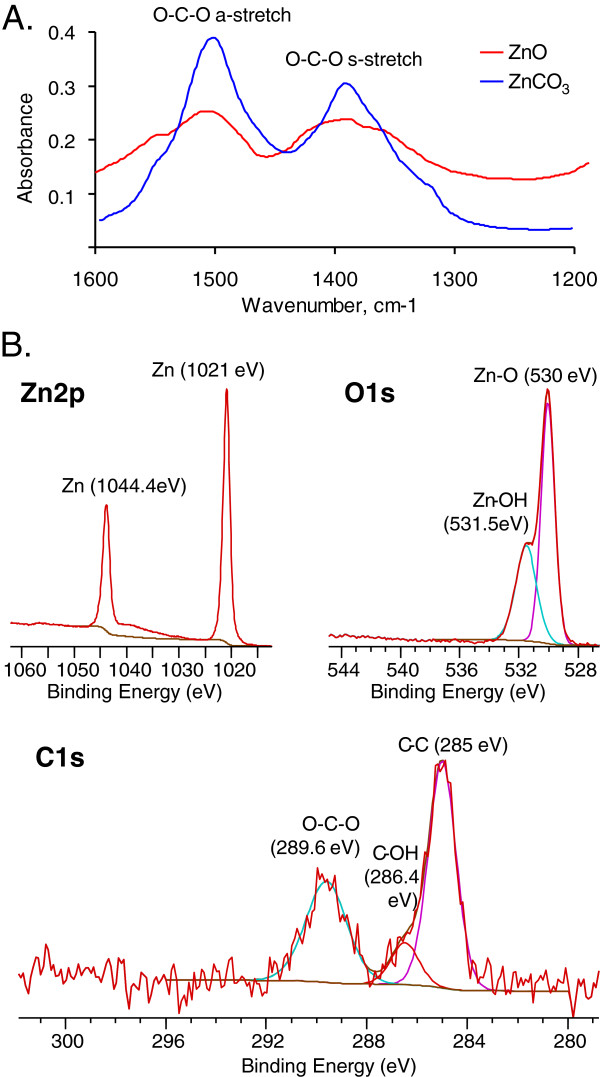
**Characterization of surface composition for commercially manufactured ZnO NPs using spectroscopic probes. A.** ATR-FTIR spectra of ZnO NPs compared to ZnCO_3_ in the 1200 t0 1600 cm^−1^ region. **B.** High resolution XPS data of ZnO NPs in the Zn 2p, O 1s and C 1s binding energy regions.

The specific surface area of the sample was calculated in the nitrogen relative pressure range from 0.05 to 0.3 where the BET isotherm is most linear. This yielded a surface area of 47 ± 1 m^2^/g for ZnO NPs for the batch of NPs used in sub-acute study and 15 ± 4 m^2^/g for the batch used in sub-chronic study. Surface composition examination using ATR-FTIR spectroscopy (Figure 2A) revealed that carbonates are present on the nanoparticle surface. XPS was also used to characterize the surface composition of the ZnO NPs. High resolution XPS spectra (Figures 2B) show binding energy peaks in the Zn 2p, C 1s, and O 1s regions of the photoelectron spectrum. The Zn 2p peaks at 1021.1 eV and 1044.4 eV (Figure 2B) can be assigned to Zn^2+^ in ZnO crystal [34]. The O 1s peak positions at 530 eV and 531.5 eV (Figure 2B) correspond to surface and near surface oxygen in crystal lattice and O–H groups on the surface [35]. The C 1s region has three peaks (Figure 2). The peak at 285.0 eV is due to adventitious carbon on the nanoparticle surfaces. Two species, C–OH (hydroxyl) and C–O–C (ether), can contribute to the 286.4 eV peak [36] and the peak at 289.6 eV is due to O–C–O (carbonate) [37].

### Dissolution of ZnO in artificial lung fluids

**Figure 3:**
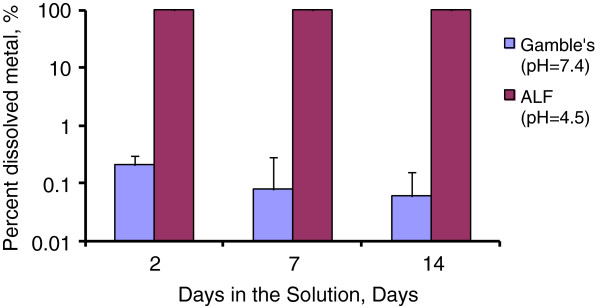
**Dissolution of ZnO NPs in simulated biological fluids, Gamble’s (pH 7.4) and ALF (pH 4.5) solutions.** The Y-axis is plotted on a logarithmic scale for clarity.

Dissolution is a surface phenomenon and the propensity of ZnO nanoparticles to dissolve into ions was investigated. As shown in Figure 3, less than 1% of ZnO dissolved in Gamble’s buffer after 2 wks. In contrast, 100% of the ZnO dissolved within the first 24 hr of mixing in ALF solution. ALF buffer contains 0.11 M concentration of citric acid, which simulates *in vivo* protein binding. Thus this result agrees with what is known about citric acid and the ability for it to adsorb to the surface thereby promoting dissolution of ZnO [23]. Gamble’s solution contains a much smaller amount of sodium citrate (0.4 mM) and a complex mixture of other anions and cations that could prevent NPs from releasing Zn^2+^, or could trap the released ions if other precipitates form. The presence of a significant amount of citrate ions (or protein binding) affects the fate and possible toxic effect of this nanomaterialas has been extensively discussed in the literature [38]. This should be taken into account when evaluating the toxicity of ZnO NPs.

### Aerosol characterization

**Figure 4:**
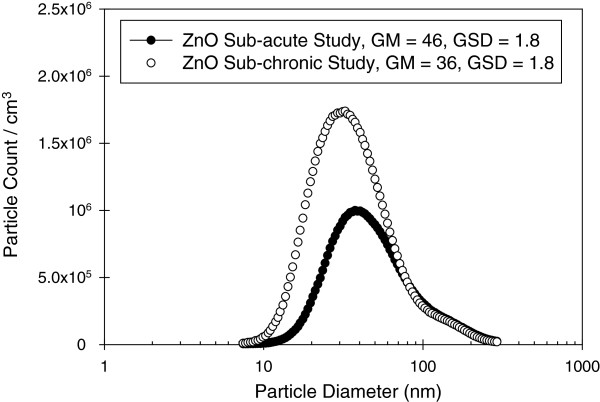
Particle size distributions of generetated ZnO aerosol measured by SMPS in sub-acute and sub-chronic studies.

ZnO nanoparticle aerosols collected in the exposure chamber were imaged using TEM and size distributions were measured using a SMPS (Figure 4). It can be seen that these substrate-collected aerosols contain primary particles of ZnO that are aggregated to form the larger aerosol. The aerosol size distribution measured in the whole-body inhalation chamber yielded a geometric mean mobility diameter of 46 nm (GSD = 1.8) and 36 nm (GSD = 1.8) in sub-acute and sub-chronic studies, respectively (Table 1). These measurements show that the mice were exposed to aggregates in the nanoscale regime below 100 nm. TEM-EDS analyses of particles deposited on the grids placed in the exposure chamber confirmed the presence of elemental Zn (data not shown).

### Dosimetry of ZnO NPs in the blood, BAL fluid and selected tissues

In the sub-acute study, Zn lung concentrations measured by ICP-MS were significantly (*p* < 0.02) higher in mice necropsied immediately (0 wk) after the exposure (144.8 ± 6.9 μg/g lung (dry wt)) than in sham-exposed mice (123.3 ± 2.6 μg/g lung (dry wt)). Concentration of Zn in the lungs of mice necropsied after the 3-wk rest period (3 wks) post exposure was 131.4 ± 13.7 μg/g lung (dry wt) (Table 2 ). Zn^2+^ concentrations in BAL fluid were significantly (*p* < 0.001) elevated immediately after the exposure (73 ± 9 μg/L) compared to sham-exposed (16 ± 2 μg/L) and mice rested for 3 wks (16 ± 2 μg/L) (Table 2). Blood, liver, spleen, kidney, heart and brain did not show any significant increase of Zn concentration with the exposure.

**Table 2:** Zn concentration in BAL fluid and organs of shams and ZnO NP-exposed mice determined by ICP-MS

	**Sub-acute study**			**Sub-chronic study**		
**Zn concentration**	**Experimental group:**			**Experimental group:**		
	**Shams (n = 6)**	**ZnO 0 wk (n = 7)**	**ZnO 3 wks (n = 6)**	**Shams (n = 6)**	**ZnO 0 wk (n = 8)**	**ZnO 3 wks (n = 6)**
BAL fluid, μg/L #	16 ± 2	73 ± 9***	16 ± 2	48 ± 10	80 ± 23	42 ± 1
Blood, μg/L	4800 ± 123	4650 ± 131	4840 ± 157	5117 ± 189	5567 ± 42	4950 ± 250 ##
Lung, μg/g (dry wt)	123.3 ± 2.6	144.8 ± 6.9**	131.4 ± 13.7	109.7 ± 8.8	102.1 ± 4.4	109.6 ± 4.3
Heart, μg/g (dry wt)	121.8 ± 2.7	127.8 ± 6.0	112.6 ± 10.1	115.4 ± 4.4	150.9 ± 14.7*	124.1 ± 7.0
Liver, μg/g (dry wt)	155.5 ± 5.5	161.3 ± 4.4	153.2 ± 10.4	100.2 ± 5.3	111.2 ± 6.0	108.9 ± 8.4
Spleen, μg/g (dry wt)	108.6 ± 2.5	114.6 ± 4.2	111.6 ± 3.9	164.9 ± 33.6	277.6 ± 46.2	156.0 ± 23.3
Kidney, μg/g (dry wt)	97.8 ± 8.4	88.0 ± 4.0	85.9 ± 5.8	96.8 ± 7.9	110.8 ± 9.9	97.2 ± 7.0
Brain, μg/g (dry wt)	101.7 ± 4.4	102.7 ± 4.1	104.7 ± 4.1	47.9 ± 3.6	43.6 ± 2.6	43.6 ± 2.7

Values are expressed as mean and SE; *p < 0.05, **p < 0.05, ***p < 0.001 significantly increased from shams.

# BAL was not digested, Zn concentration represents mainly dissolved Zn ions in BAL fluid.

## n = 3.

In the sub-chronic study, Zn concentrations in BAL fluid, blood, spleen, and kidneys were elevated in the group sacrificed immediately post exposure compared to controls, but this increase was not statistically significant (Table 2). Zinc in hearts in the group at 0 wk post exposure was significantly (*p* < 0.05) increased compared to shams. Concentrations in other major organs did not differ from sham-exposed mice.

### Animal weight gain

**Figure 5:**
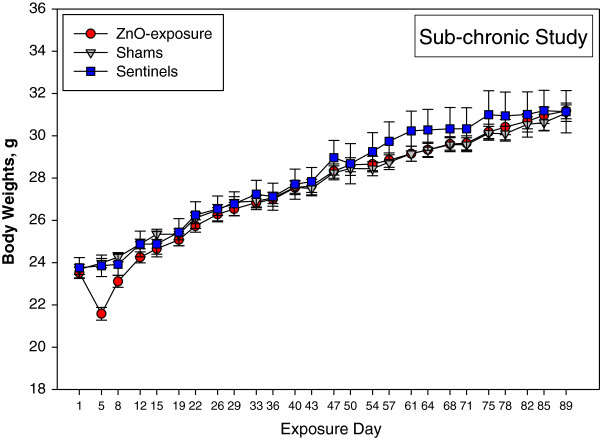
**Body weights of animals during sub-chronic exposure to ZnO NPs.** There was a significant decline in the body weights of mice on day 5 of ZnO NP exposure, compared to controls or sentinels.

We observed a body weight loss in mice (average of 2 g) after the first week of exposure in the sub-chronic ZnO NP study (Figure 5). This drop was not observed in the sub-acute study, in which mice gained 0.6 g after the first week of exposure. Animals exposed to laboratory air and sentinels gained 0.8 g and 0.9 g, respectively.

### Evaluation of BAL fluid and hematology parameters

#### Sub-acute study

**Figure 6:**
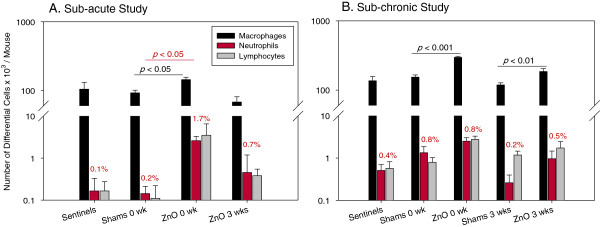
**Number of differential cells in BAL fluid in controls and animals exposed to ZnO NPs for period of 2 (A) or 13 wks (B).** In both studies there was a significant increase in number of macrophages in BAL fluid, in comparison with shams necropsied at the same time after exposure. The number of neutrophils was significantly increased only in sub-acute study, however biologically this increase was just marginal. Data are expressed as mean ± SE.

The number of total cells in BAL fluid (Table 3) was significantly (*p* < 0.01) increased in mice exposed to ZnO NPs and necropsied at 0 wk post exposure (150.3 ± 14.9 × 10^3^ cells/mouse) as compared to control mice (92.7 ± 8.6 × 10^3^ cells/mouse). Similarly, as shown in Figure 6A, the number of macrophages (144.3 ± 11.8 × 10^3^ cells/mouse) was significantly (*p* < 0.01) increased compared to shams (92.5 ± 8.6 x 10^3^ cells/mouse). Mice necropsied at 0 wk post exposure had significantly higher (*p* < 0.02) recruitment of neutrophils (2.6 ± 0.7 × 10^3^ cells/mouse) as compared to shams (0.1 ± 0.07 × 10^3^ cells/mouse). However, the proportion of neutrophils among total cells was only 1.7% in mice necropsied at 0 wk.

**Table 3:** Total number of cells, total protein concentration and activity of LDH in BAL fluid in shams and ZnO NP-exposed mice (sub-acute study)

**Sub-acute study**		**Experimental group:**		
**Parameter in BAL fluid**	**Sentinels**	**Shams**	**ZnO**	**ZnO**
	**(n = 2)**	**0 wk**	**0 wk**	**3 wks**
		**(n = 6)**	**(n = 6)**	**(n = 6)**
Total cells/mouse, n × 10^3^	105.0 ± 27.0	92.7 ± 8.6	150.3 ± 14.9**	67.0 ± 11.2
Total protein, μg/mL	122.2 ± 2.9	135.5 ± 6.8	125.5 ± 4.1	131.3 ± 5.8
LDH activity, U/L	63 ± 18	46 ± 4	32 ± 9	93 ± 23**

Values are expressed as mean and SE; **p < 0.01 significantly different from sham-exposed animals (one way ANOVA).

Concentrations of total protein analyzed in BAL fluid were not different between ZnO NP-exposed groups and controls. The activity of LDH was not different between mice necropsied at 0 wk and shams but was significantly higher 3 wks post exposure (*p* < 0.01, Table 3).

**Figure 7:**
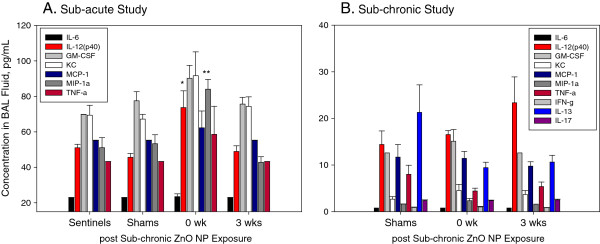
**Concentration of selected cytokines/chemokines in BAL fluid of mice exposed sub-acutely (A) or sub-chronicly (B) to ZnO NPs.** Data are expressed as mean ± SE; *p < 0.05, **p < 0.01 significantly different from shams.

Only 2 of the 7 measured inflammatory cytokines/chemokines were significantly elevated at 0 wk post exposure, IL-12p(40) and MIP-1α (p < 0.05 and p < 0.01, respectively). These concentrations were increased 1.6- and 1.3-fold from levels in sham-exposed mice (Figure 7A). Hematology parameters were evaluated to assess if exposure to ZnO NPs caused any systemic inflammatory changes (Table 4). We found no significant differences between ZnO NP-exposed mice and shams, with the exception of hematocrit that was significantly higher in mice exposed to ZnO NPs and necropsied 3 wks post exposure compared to shams (52.7% vs. 47.7%, respectively).

**Table 4:** Blood parameters in shams and mice after sub-acute exposure to ZnO NPs

**Sub-acute study**	**Experimental group:**		
**Whole blood parameter**	**Shams**	**ZnO 0 wk**	**ZnO 3 wks**
WBC × 10^3^/μL	2.91 ± 0.9	2.81 ± 0.3	3.11 ± 0.4
RBC × 10^6^/μL	9.85 ± 0.3	10.27 ± 0.5	9.49 ± 0.2
Hemoglobin, g/dL	16.5 ± 0.4	17.3 ± 0.6	15.3 ± 0.2
Hematocrit,%	47.7 ± 1.3	51.3 ± 2.0	52.7 ± 1.1**
Monocytes × 10^3^/μL	0.33 ± 0.1	0.33 ± 0.0	0.39 ± 0.1
Neutrophils × 10^3^/μL	0.56 ± 0.1	0.38 ± 0.1	0.84 ± 0.1
Lymphocytes × 10^3^/μL	1.95 ± 0.7	2.02 ± 0.3	1.83 ± 0.3
Eosinophils × 10^3^/μL	0.052 ± 0.016	0.052 ± 0.016	0.082 ± 0.019
Basophils × 10^3^/μL	0.014 ± 0.008	0.022 ± 0.009	0.018 ± 0.007

Values are expressed as mean and SE, WBC - white blood cells, RBC - red blood cells; **p < 0.02 (significantly different from shams), n = 6 in each group.

#### Sub-chronic study

There was a significant enhancement in the recruitment of cells to the lungs after sub-chronic exposure to ZnO NPs compared to sham-exposed mice. Mice euthanized immediately after the last ZnO NP exposure had a 2-fold higher number of cells in BAL fluid than shams (Table 5). This increased cellularity was represented mainly by macrophages, and there were no significant increases in numbers of neutrophils or lymphocytes between ZnO NP-exposed mice and their sham-exposed counterparts (Figure 6B). The concentration of total protein and activity of LDH in BAL fluid were not different between ZnO NP-exposed mice and their controls (Table 6). In the sub-chronic study we also measured the concentration of MDA in homogenates of lung tissue as a marker of ROS generation and found no significant difference between exposed and control mice. Whole blood parameters did not show any sign of systemic inflammatory response (Table 6). Similarly like in sub-acute study, hematocrit in mice exposed for 13 wks sacrificed at 3 wks post exposure was increased. Cytokine/chemokine concentrations in BAL fluid for the sub-chronic study are shown in Figure 7B. The results show no significant differences in ZnO NP-exposed groups and shams. The concentrations of IL-4 and IL-5 were below lowest limit of detection (2.20 pg/mL and 5.38 pg/mL, respectively).

**Table 5:** Total number of cells, total protein concentration and activity of LDH in BAL fluid and MDA concentration in the lungs in shams and ZnO NP-exposed mice (sub-chronic study)

**Sub-chronic study**		**Experimental group:**			
	**Sentinels**	**Shams**	**ZnO**	**Shams**	**ZnO**
		**0 wk**	**0 wk**	**3 wks**	**3 wks**
Parameter in BAL fluid (n = 6):					
Total cells/mouse, n × 10^3^	137.3 ± 20.5	156.0 ± 11.8	302.0 ± 11.5***	116.3 ± 8.3	188.0 ± 19.3**
Total protein, μg/mL	101.7 ± 3.0	101.8 ± 2.4	108.4 ± 5.5	117.5 ± 16.2	123.8 ± 6.6
LDH activity, U/L	106.0 ± 19.5	60.8 ± 14.6	74.7 ± 12.7	95.3 ± 12.1	83.7 ± 14.5
Parameter in Lung (n = 6):					
MDA concentration, nmol/mL	NA	50.0 ± 2.5	58.7 ± 6.8	NA	NA

Values are expressed as mean and SE; ***p < 0.001, **p < 0.01 significantly different from control counterparts (one way ANOVA followed by Tukey test); NA not analyzed.

**Table 6:** Blood parameters in shams and mice after sub-chronic exposure to ZnO NPs

**Whole blood parameter**		**Experimental group:**		
	**Sentinels**	**Shams 0 wk**	**ZnO 0 wk**	**ZnO 3 wks**
WBC × 10^3^/μL	3.62 ± 0.5	4.18 ± 0.6	3.79 ± 1.0	4.90 ± 0.4
RBC × 10^6^/μL	9.66 ± 0.2	9.20 ± 0.2	9.6 ± 0.4	9.63 ± 0.3
Hemoglobin, g/dL	15.4 ± 0.2	16.0 ± 0.2	16.2 ± 0.4	16.2 ± 0.2
Hematocrit,%	50.2 ± 0.7	45.0 ± 0.6	47.8 ± 0.3	52.9 ± 1.7
Monocytes × 10^3^/μL	0.34 ± 0.1	0.22 ± 0.1	0.24 ± 0.1	0.20 ± 0.3
Neutrophils × 10^3^/μL	2.11 ± 0.5	0.92 ± 0.2	1.29 ± 0.3	1.9 ± 0.2
Lymphocytes × 10^3^/μL	1.14 ± 0.2	2.98 ± 0.4	2.20 ± 0.9	2.75 ± 0.4
Eosinophils × 10^3^/μL	0.032 ± 0.010	0.055 ± 0.013	0.062 ± 0.035	0.074 ± 0.013
Basophils × 10^3^/μL	0.002 ± 0.001	0.013 ± 0.003	0.013 ± 0.004	0.008 ± 0.003

Values are expressed as mean and SE, WBC - white blood cells, RBC - red blood cells, n = 6 in each group.

### Lung histopathology in sub-acute and sub-chronic study

**Figure 8:**
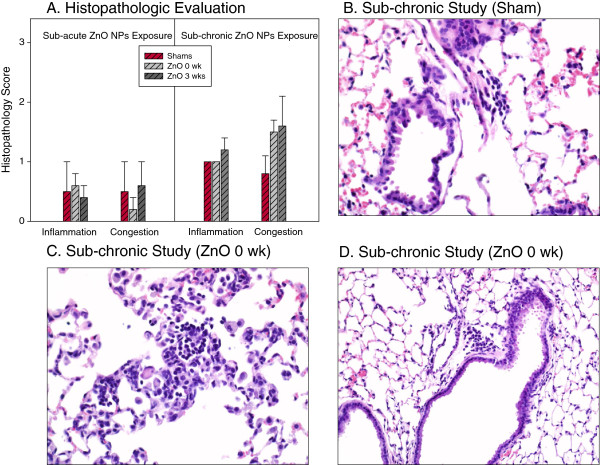
**Qualitative histopathological evaluation and micrographs of lung tissues of mice exposed to ZnO NPs.** Histopathologic evaluation (focusing on inflammation, cell injury, congestion, fibrosis and eosinophilia) was performed by light microscopy using a semi-quantitative 4-point scale: 0 - no lesion, 1 - minor focal change (<10% of lung), 2 – multifocal to coalescing inflammation (11-50%), and 3 – widespread > 50% **(A)**. Micrographs of lung tissues showing most common minor changes: focal perivascular lymphoid cells (40x, **B**), focal accumulation of mononuclear cells (40x, **C**), and minor perivascular lymphoid cells (20x, **D**).

Since a preliminary bright field microscopy screening of lung tissues with H&E staining showed a lack of evidence for overt lesions, a more sensitive scale for the detection of changes in pathologic parameters (inflammation, cell injury, fibrosis, congestion and possible eosinophila) was imposed. Due to application of this 4-point scale (0 - no lesion, 1 - minor focal change [<10% of lung], 2 – multifocal to coalescing inflammation [11-50%], and 3 – widespread > 50%), even the control animals showed presence of many evaluated histology parameters, indicating that the extent of lesions found in exposed animals was not due to ZnO NP exposure. In both, the sub-acute and sub-chronic study, there were a few lungs that showed a slight increase in number of macrophages and/or with occasional evidence of alveolar macrophage activation (slightly foamy cytoplasm, karyomegaly, binucleate, and mitotic figures). In addition to these changes, rare minor foci of perivascular lymphoid cells and/or focal septal hypercellularity were found in sub-chronic study mice. There was a distinct lack of cell injury, fibrosis or eosinophil infiltration in mice from either study. In the few sites with hypercellularity of the alveolar septa, there were occasional granulocytes partially sequestered in the septa composed of both neutrophils and eosinophils, however these were just marginal inside of sticky capillaries due to minor focal activation. Figure 8A shows the histopathology scores from the lung tissues. Most common minor changes found in lung tissues of controls or ZnO NP-exposed mice are shown in Figures 8B, C and D.

### Pulmonary mechanics measurements in sub-acute and sub-chronic study

**Figure 9:**
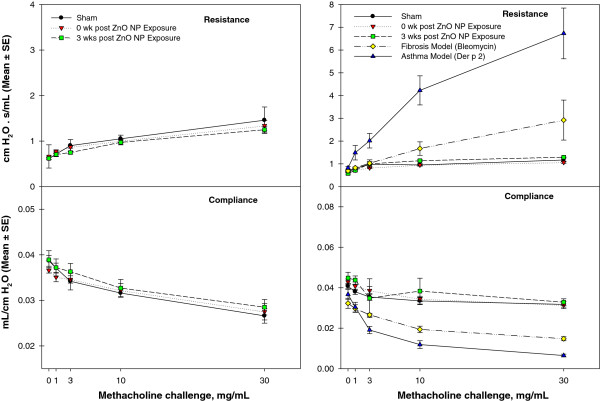
**Results of pulmonary mechanics measurements of mice after sub-acute (A) or sub-chronic (B) exposure to ZnO NPs or filtered laboratory air (sham).** Resistance and compliance were measured after inhalation challenge to increasing concentration of methacholine (1, 3, 10, and 30 mg/mL). Plot B also shows resistance and compliance in positive control mice exposed by nasal instillation to bleomycin (fibrosis model) or house dust mite allergen (*Dermatophagoides pteronyssinus*, asthma model). No changes in pulmonary mechanics were observed in mice exposed to ZnO NPs for 2 **(A)** or 13 wks **(B)**.

Measurements of pulmonary mechanics involving resistance and compliance without cholinergic agonist showed no difference between mice exposed to ZnO NPs and shams-exposed mice (Figure 9). Treatment with escalating doses of methacholine showed no evidence of hyperreactive airways due to exposure to ZnO NPs. For comparison, positive control mice treated with bleomycin *via* intra-tracheal instillation (to induce transient fibrosis) or mice sensitized and challenged with house dust mite allergen (*Dermatophagoides pteronyssinus,* Der p 2) demonstrated increasing resistance and decreasing compliance with increasing doses of inhaled methacholine. We conclude that inhalation exposure to ZnO NPs for period of 2 or 13 wks did not cause clinically significant remodeling of the airway in mice nor did it induce hyperreactive airways.

## Discussion

Our studies assessed toxicological effects of ZnO NPs in mice following sub-acute (2-wk) and sub-chronic (13-wk) inhalation exposure. Nanoparticles were fully characterized prior to and during exposures. Geometric mean mobility diameter of ZnO NP aerosol sampled from the whole-body chamber was 46 nm and 36 nm in sub-acute and sub-chronic study, respectively and demonstrated a moderate degree of aggregation.

The bioavailability and toxicity of metal oxide nanoparticles are often related to the ability of the nanoparticles to deliver soluble metal ions to the adjacent tissues [22]. Nanoparticles often have enhanced dissolution kinetics due to their larger surface area in comparison with bulk material [23]. Additionally, the extent of dissolution can be size dependent [23]. Multiple studies have shown that ZnO NPs dissolve in the acidic environment of the phagolysosomes [22,26]. Here, we observed ligand-promoted dissolution [39] facilitated by citrate ligands used in ALF solution to represent a coordinating moiety that might be found in protein binding as well (Figure 3). Our data suggest that ZnO NPs dissolved in the lung (Table 2). Concentration of Zn^2+^ in the supernatants of BAL fluid was 4.5-fold higher in mice necropsied immediately after the exposure than in shams or mice necropsied 3 wks post exposure (Table 2). For the sub-chronic study (Table 2), the increase of Zn^2+^ ions in BAL fluid from shams was lower, the concentration was only 2-fold higher than in shams or mice at 3 wks post exposure. However, this increase was not statistically significant (p = 0.065). Interestingly, in the sub-chronic study, we did not find any increase in Zn in the lungs of mice sacrificed at 0 wk in comparison with controls. Evaluation of BAL cells and lung tissues using dark field microscopy as well as TEM-EDS did not show a distinct presence of Zn NPs inside the macrophages or lung tissues (data not shown). The blood Zn concentrations were slightly elevated in mice at 0 wk in sub-chronic study, but not in sub-acute study. Since we have used a whole-body exposure chamber in our studies, it is possible that some portion of ZnO particles was ingested and transferred to the blood through the gastro-intestinal system. None of the other organs analyzed in either study had significantly elevated Zn concentrations. Wang *et al*. performed an acute instillation study in Wistar rats in which a bolus of 2.5 mg/kg ZnO NPs was delivered by a dry powder sprayer directly into nasal passages, twice daily for 3 days (total dose 15 mg/kg) [10]. Doses in our studies were lower with 2.0 mg/kg delivered in 14 days (sub-acute) and 10.9 mg/kg delivered in 89 days (sub-chronic). Even at this high dose, Wang *et al.* found no significant differences in Zn content in lungs compared to controls, Zn levels were significantly increased in livers at 12 hr and 36 hr time point and in kidneys at 36 hr time point. Furthermore, they reported that exposure caused inflammation, interstitial hyperemia, emphysema and hyperplasia in the lungs and serious hepatic lesions. These data further suggest that ZnO NPs dissolve in the lungs or translocate to the blood for circulation. Intravenously administered ZnO NPs exhibited a primary retention in lung for the first hour and were excreted *via* intestines into feces within 24 h [40]. Such fast elimination of ZnO NPs from the system might be attributed to a high dissolution of these particles in biological fluids with lower pH as shown in Figure 3. The NIEHS Nano GO Consortium found ZnO NPs to be the most cytotoxic in *in vitro* studies (out of three forms of TiO_2_, and three forms of multiwalled carbon nanotubes [MWCNTs] [41]. Unfortunately, ZnO NPs were not included in their *in vivo* interlaboratory evaluation and hence provide no basis for comparison with this study [42].

ZnO dissolution is relevant to pulmonary toxicity due to inhalation exposure to ZnO in the welding fumes in occupational settings [43] as well as for use of ZnO as a raw material in manufacturing. Inhalation of ultrafine ZnO was found to be the major cause of zinc fume fever that is characterized by increased neutrophils in BAL fluid, along with an increase in TNF-α, IL-1, and IL-8, followed by decreased lung function, cough, fever, and metallic taste [43]. The National Institute for Occupational Safety and Health (NIOSH) has established a recommended exposure limit for ZnO of 5 mg/m^3^ as a time-weighted average concentration for up to a 10-hr workday and a 40-hr workweek. The estimated doses of ZnO NPs in tracheobronchial and pulmonary region of mouse were 51 μg/mouse (2.0 mg/kg) in the sub-acute and 306 μg/mouse (10.9 mg/kg) in the sub-chronic study. These estimated doses correspond to the lung dose accumulated by a 70 kg person exposed to a concentration of 5 mg/m^3^ for 13 or 71 workdays (assuming breathing frequency 15 breaths/min, 600 mL/breath, and pulmonary deposition fraction for 40 nm particles of 0.5 [31].

Our previous toxicity studies of copper, iron or silver NPs [28,29,44] are in agreement with “the higher solubility - the higher toxicity effect” [11,45]; however ZnO NPs despite very high solubility in acidic milieu that contain coordinating ligands demonstrated minimal toxicity in our inhalation studies. Of the many pulmonary biomarkers measured, we observed biologically significant increase only in total number of cells in BAL fluid mainly due to an increase in macrophages and a moderate increase in IL-12(p40) and MIP-1α in BAL fluid. Measurements of a cytototoxicity biomarker (activity of LDH) in BAL fluid showed a significant increase in mice sacrificed at 3 wks post sub-chronic exposure compared to controls. ROS in the lungs (assessed *via* MDA) were not different in ZnO NP-exposed mice and controls. Histopathology evaluation of lung tissues as well as pulmonary mechanics measurements revealed no significant changes from controls. There were no eosinophils and few neutrophils found in BAL fluid or lung tissue in mice after inhalation exposure to ZnO NPs in sub-acute or sub-chronic exposure. Furthermore, the concentrations of IL-4, IL-5, IL-13 and IL-17 in BAL fluid were not elevated after exposure to ZnO NPs. Our results are contrary to data reported by Cho *et al.*[8,26,46]. Intra-tracheal instillation of ZnO NPs at a surface area dose of 50 or 150 cm^2^/rat (corresponding to a mass of 0.5 mg/kg or 1.4 mg/kg, respectively) caused eosinophilia, proliferation of airway epithelial cells, goblet cell hyperplasia, and pulmonary fibrosis. This eosinophilic response in BAL fluid was not reproduced in mice (C57Bl/6 and BALB/c) after an aspiration of ZnO NPs dispersed in 5% heat-inactivated mouse serum (15 cm^2^/mouse or 1.2 mg/kg), but these authors reported that the concentration of eotaxin and IL-13 in the BAL fluid was significantly increased at 24 hr post exposure compared to controls [26]. Later studies by this group found that recruitment of eosinophils was initiated by metal oxide nanoparticles themselves not their soluble ions [46]. To assess this discrepancy, we exposed mice to ZnO NPs by intra-tracheal instillation (4 mg/kg or 47 cm^2^/mouse) with necropsy 24 hr after the exposure and found a higher recruitment of neutrophils (40%) in BAL fluid (data not shown) than in shams (2%) in which no eosinophils were found. The recruitment of neutrophils to the lungs in case of instillation exposure might be a part of an acute inflammatory response to a different exposure method (instillation *vs.* inhalation). Our studies confirm that ZnO NPs have very modest inflammatory potency *in vivo* after repeated inhalation exposures but one time instillation exposure of a bolus dose of NPs caused an inflammatory response. These results support the notion that *in vitro* ZnO NP studies in submersed condition may produce false-positive results due to a higher dissolution of ZnO NPs in media without further translocation and clearance. Furthermore, Cho *et al*. [26] observed that large poorly dispersed agglomerates of ZnO NPs induced lower eosinophilia (1%) compared to well-dispersed ZnO NPs (38%) and hypothesized that eosinophilic inflammation might be induced only by well dispersed ZnO NPs at high doses. We suggest that inhalation studies at environmentally relevant doses represent more realistic exposures to ZnO NP aerosols.

In our studies, we found a significant but modest increase in IL-12(p40) as well as MIP-1α compared to controls after sub-acute exposure to ZnO NPs. Both cytokines are released by activated macrophages [47]. Histopathology evaluation of lung tissues showed a moderate presence of slightly foamy alveolar macrophages in mice exposed to ZnO NPs necropsied immediately post exposure. On the other hand, this finding might be important for a possible use of ZnO NPs in an anti-cancer treatment. It was reported that ZnO NPs induce release of the proinflammatory cytokines (IL-12, IFN-γ, and TNF-α) without major cytotoxicity effects [48] and our studies support this potential use.

Development of clinical tolerance to repeated inhalation exposure to ZnO fumes from welding processes has been reported in clinical studies of metal fume fever [5] as well as in a study of zinc foundry workers [49] and a studies of ZnO fumes in mice [6,50]. Symptoms of metal fume fever usually dissipate within 24–48 hr without major lung injury [43,49]. It is possible that repeated inhalation exposure to ZnO NPs at low exposure concentrations may lead to the attenuation of pulmonary responses. In our sub-chronic study, we observed a decrease in body weight of animals after first 5 days of exposure to ZnO NPs (Figure 5) which might be a clinical sign of initial response to ZnO NPs that was later diminished with repeated exposures. In the pulmonary tolerance study [6], NIH-Swiss mice were exposed to 1 mg/m^3^ ZnO for 1, 3, or 5 days, mice acquired tolerance to neutrophil recruitment to the lungs, however tolerance to total protein in BAL fluid was not observed suggesting persistence of lung injury. Furthermore, a greater lung pathology was found in mice exposed repeatedly to ZnO compared to the ones exposed one time. Development of pulmonary tolerance to repeated exposure of inhaled toxicants is not fully understood and more studies are necessary to confirm this phenomenon.

## Conclusions

The results of *in vitro* and *in vivo* studies of ZnO NP toxicity have been contradictory and thus controversial. Our sub-acute (2-wk-) and sub-chronic (13-wk-) whole-body inhalation exposure studies with average ZnO NP concentration of 3.5 mg/m^3^ induced a significant increase in recruitment of total white blood cells to the lungs that was represented mainly by increased macrophages and a moderate increase of IL-12 (p40) and MIP-1α. None of the other inflammatory pulmonary markers measured in BAL fluid or lungs, histopathology evaluation or changes in pulmonary mechanics after methacholine challenge were significantly different from sham-exposed controls. We observed slightly elevated hematocrit values at 3 wks post exposure in both sub-acute and sub-chronic exposure. Our study suggests a high dissolution of ZnO NPs in lung tissues and translocation of Zn to the blood circulation. Furthermore, our study shows that the higher dissolution of metal-based nanomaterials is not necessarily associated with higher toxicity upon inhalation exposure as seen here for ZnO.

## Abbreviations

Ag: Silver; ALF: Artificial lysosomal fluid; ANOVA: Analyses of variance; AMTIR: Amorphous material transmitting infrared radiation; ATR: Attenuated total reflectance; BAL: Bronchoalveolar lavage; C: Dynamic Compliance; CeO2: Cerium dioxide; Derp: *Dermatophagoides pteronyssinus*; EDS: Energy dispersive spectrometer; FTIR: Fourier transform infrared; GM: Geometric mean; GM-CSF: Granulocyte macrophage colony stimulating factor; GSD: Geometric standard deviation; H & E: Hematoxylin and eosin; HEPA: High-efficiency particulate air; ICP-OES: Inductively coupled plasma - optical emission spectroscopy; IL: Interleukin; IFN: Interferon; KC: Keratinocyte-derived cytokine; LDH: Lactate dehydrogenase; MCP: Monocyte chemotactic protein; MDA: Malondialdehyde; MIP: Macrophage inflammatory protein; NIOSH: National Institute for Occupational Safety and Health; NIEHS: National Institute of Health Sciences; ICP-MS: Inductively coupled plasma-mass spectrometry; MWCNTs: Multiwalled carbon nanotubes; NPs: Nanoparticles; R: Dynamic resistance; RAW 264.7: Mouse leukemic monocyte macrophage; ROS: Reactive oxygen species; SMPS: Scanning mobility particle sizer; TBARS: Thiobarbituric acid reactive substances; TEM: Transmission electron microscopy; TiO2: Titanium dioxide; TNF: Tumor necrosis factor; XPS: X-ray photoelectron spectroscopy; XRD: Powder X-ray diffraction; ZnO: Zinc oxide.

## Competing interests

The authors (AA-D, LVS, JSK, SUV, APA, PTO, VHG and PST) report no competing interests.

## Authors’ contributions

AA-D designed animal studies, conducted animal exposures, biological assays, pulmonary mechanics measurements and analyses, elemental analyses in mouse tissues, processed particle size distribution data, data analysis, and drafted the manuscript. LVS performed nanomaterial characterization, dissolution studies, elemental analyses, and assisted with manuscript drafting. JSK participated in the study design, conducted animal exposures, necropsies and biological assays. SUV assisted with animal exposures, necropsies and biological assays in the sub-acute study. APA assisted with nanomaterial characterization. PTO developed and coordinated the aerosol generation system for inhalation exposures and characterized exposure concentrations and aerosol size distribution. VHG initiated the integrated study design, led analyses of nanomaterial characterization and dissolution data, authored portions of the manuscript and edited the final manuscript. PST designed the animal studies, coordinated the biological assays and data analysis, and authored the final manuscript. All authors read and approved the final manuscript.
